# Valorization of Cashew Apple Waste into a Low-Alcohol, Healthy Drink Using a Co-Culture of *Cyberlindnera rhodanensis* DK and *Lactobacillus pentosus* A14-6

**DOI:** 10.3390/foods13101469

**Published:** 2024-05-09

**Authors:** Nang Nwet Noon Kham, Somsay Phovisay, Kridsada Unban, Apinun Kanpiengjai, Chalermpong Saenjum, Saisamorn Lumyong, Kalidas Shetty, Chartchai Khanongnuch

**Affiliations:** 1 Multidisciplinary School, Chiang Mai University, Muang, Chiang Mai 50100, Thailand; nwenoonkham@gmail.com (N.N.N.K.); somsay2009@hotmail.com (S.P.); 2 Division of Food Science and Technology, Faculty of Agro-Industry, Chiang Mai University, Mae-Hia, Chiang Mai 50100, Thailand; 3 Department of Chemistry, Faculty of Science, Chiang Mai University, Huay Kaew Rd., Muang, Chiang Mai 50200, Thailand; apinun.k@cmu.ac.th; 4 Faculty of Pharmacy, Chiang Mai University, Muang, Chiang Mai 50200, Thailand; chalermpong.s@cmu.ac.th; 5 Department of Biology, Faculty of Science, Chiang Mai University, Huay Kaew Rd., Muang, Chiang Mai 50200, Thailand; saisamorn.l@cmu.ac.th; 6 Global Institute of Food Security and International Agriculture (GIFSIA), Department of Plant Sciences, North Dakota State University, Fargo, ND 58108, USA; kalidas.shetty@ndsu.edu; 7 Research Center for Multidisciplinary Approaches to Miang, Multidisciplinary Research Institute (MDRI), Chiang Mai University, Muang, Chiang Mai 50200, Thailand; 8 Research Center of Microbial Diversity and Sustainable Utilization, Chiang Mai University, Huay Kaew Rd., Muang, Chiang Mai 50200, Thailand

**Keywords:** *Cyberlindnera rhodanensis*, *Lactobacillus pentosus*, low-alcohol beverages, co-culture fermentation, cashew apple waste, healthy drinks

## Abstract

This study investigated the potential of microbial fermentative transforming processes in valorizing the cashew apple by-product into a low-alcohol, health-benefiting beverage. We particularly investigated the use of a non-*Saccharomyces* yeast, *Cyberlindnera rhodanensis* DK, as the main targeted microbe. At 30 °C without agitation, *C. rhodanensis* DK caused changes in key parameters during the fermentation of cashew apple juice (CAJ) in terms of varied pH values and initial sugar concentrations. This result indicated that pure CAJ, with pH adjusted to 6 and with the original 6.85% (*w*/*v*) total sugar content, was the most feasible condition, as glucose and fructose were mostly consumed at 12 days of fermentation. A co-culture approach with either *Saccharomyces cerevisiae* TISTR 5088 or *Lactobacillus pentosus* A14-6 was investigated to improve both physicochemical and fermentation characteristics. Co-fermentation with *S. cerevisiae* TISTR 5088 resulted in significantly increased ethanol accumulation to 33.61 ± 0.11 g/L, but diminished bioactive compounds, antioxidant activity, and antidiabetic potential. In contrast, co-fermentation with *L. pentosus* A14-6 demonstrated excellent outcomes, as it significantly increased sugar consumption and finally remained at only 4.95 g/L compared to *C. rhodanensis* DK alone, produced lower levels of ethanol at only 19.47 ± 0.06 g/L, and higher total titratable acid (TTA), resulting in a final pH of 3.6. In addition, co-fermentation with this lactic acid bacterium significantly enhanced bioactive compounds and antioxidant activity and also retained potential antidiabetic properties. These findings highlight the feasibility of using tailored microbial fermentation strategies to produce low-alcohol beverages with enhanced health-promoting properties from CAJ; however, product-development processes following health food regulations and sensory evaluation are necessary.

## 1. Introduction

Cashew apples are often recognized as an agricultural by-product and waste in the cashew industry during cashew nut processing. The relative neglect of the cashew apple in commercial terms, compared to the nuts, can largely be attributed to its bitter and astringent taste, as well as its high perishability [1]. However, cashew apples are a rich source of bioactive secondary metabolites including polyphenols such as flavonoids, anacardic acids, tannins, and carotene [2]. Furthermore, they boast a variety of nutritional components, such as vitamins (ascorbic acid, thiamine, niacin, riboflavin), organic acids (malic acid and citric acids), and significant minerals, such as copper, zinc, sodium, potassium, calcium, iron, phosphorus, and magnesium, all contributing to their potential health benefits. Moreover, cashew apples contain sugars such as glucose, fructose, and sucrose, which make them suitable for alcoholic fermentation. Their sweet aroma and sharp flavor further enhance their appeal [3]. These collective attributes of cashew apples make them an excellent, cost-effective substrate for producing fermented beverages, as indicated by various studies [4]. Furthermore, cashew apples are believed to possess several advantageous properties, including antibacterial, antifungal, anticancer, antioxidant, and antimutagenic effects [5]. Globally, cashew apples are processed into various products, including juice, syrup, jam, ice cream, candy, chutney, pickles, and others [2]. Researchers have successfully processed cashew apples for producing wine, low-alcohol fermented beverages, and bioethanol using *Saccharomyces cerevisiae* [5,6,7]. Furthermore, lactic acid bacteria have been employed to create probiotic beverages and lactic acid from cashew apples [8,9].

In the realm of alcoholic beverage production, yeasts are responsible for facilitating fermentation, in which the bioconversion of substrates into alcohol, carbon dioxide, biomass, and various volatile compounds occurs [10]. Among the arrays of yeast species, *S. cerevisiae* strains stand out as renowned ethanol producers, characterized by their rapid sugar consumption, high ethanol yield, and excellent ethanol tolerance. This specialization in metabolic pathways makes *S. cerevisiae* a reliable choice for alcohol production [11]. Non-*Saccharomyces* species contribute to significantly diversifying flavors and aromas of alcoholic beverages; however, they are often associated with substantial sugar residuals and different levels of alcohol production [12,13]. Non-*Saccharomyces* yeasts can be broadly classified into two groups; flavor-producing yeasts and neutral yeasts, which produce few or no flavor compounds. Notably, flavor-producing yeasts, such as the genera *Pichia*, *Kluyveromyces*, *Candida*, *Cyberlindnera*, and *Wikerhamomyces*, are also prolific producers of esters; therefore, non-*Saccharomyces* yeasts have proven to enhance the aromatic profile of alcoholic beverages [14,15]. Moreover, these yeasts are recognized for their capacity to produce glucosidase enzymes, which can release volatile compounds by hydrolyzing glycosidic bonds. In wine-making, this capability enables the creation of wines with more pleasant, typical, and strain-specific aroma characteristics [15]. Various yeasts of the genera *Candida*, *Kloeckera*, *Hanseniaspora*, *Zygosaccharomyces*, *Schizosaccharomyces*, *Torulaspora*, *Brettanomyces*, *Saccharomycodes*, *Pichia*, and *Williopsis* have been explored for their potential in beverage production [16]. Recent studies have highlighted that a non-*Saccharomyces* yeast, *Cyberlindnera* sp., can produce β-glucosidase that enhances aroma compounds in beverage production [17]. Kodchasee et al. [14] reported that *C. rhodanensis* produces not only β-glucosidase enzyme but also enhances the formation of esters, ethyl acetate, isoamyl acetate, and essential esters in wine production. We hypothesized that *C. rhodanensis* DK derived from fermented tea products (‘laphet’) of Myanmar is a yeast strain capable of producing β-glucosidase and therefore facilitating aroma characteristics by releasing aromatic compounds through cleavage of glycoside bonds between aroma molecules and terminal sugar [18].

Several studies have reported that non-*Saccharomyces* yeasts offer higher polysaccharide contents, richer bioactive compounds, and antioxidant capacities, as well as lower ethanol levels, contributing to the production processes of healthier low-alcohol beverages [17]. Low ethanol consumption can provide additional advantages to improve insulin sensitivity and elevate high-density lipoprotein levels, while excessive ethanol intake has been suggested to have detrimental effects on cardiovascular health, such as high blood pressure, strokes, cancer, liver disease, etc. [19]. The production of low-alcohol beverages using *C. rhodanensis* and *Wikerhamomyces anomalus* resulted in health-targeted distinctly flavored drinks with alcohol concentrations of 1.0–2.0% (*v*/*v*) [14], while Yang et al. [20] also demonstrated *Torulaspora delbrueckii* as an alternative yeast for producing low-alcohol beverages. Based on these promising studies, the utilization of non-*Saccharomyces* yeasts from laphet in the production of low-alcohol beverages holds excellent promise and is the target of this study.

This research report describes the production of a low-alcohol, healthy beverage via the fermentation process of cashew apple juice (CAJ) derived from the waste from a cashew nut-producing farm using the selected yeast strain *C. rhodanensis* DK isolated from laphet-so, a fermented tea product from Myanmar. The physicochemical characteristics of the fermentative products of *C. rhodanensis* DK monoculture and co-fermentation approaches either with the selected lactic acid bacterium *Lactobacillus pentosus* or the yeast *Saccharomyces cerevisiae* are also reported. This study also elucidates the final product assessment for potential antidiabetic activity by using in vitro assay-based models.

## 2. Materials and Methods

### 2.1. Raw Material

The cashew apples used in this study were collected from the cashew nut production process at a cashew apple cultivation farm located in Uttaradit province, Thailand. After collection, the cashew apples were thoroughly washed with clean tap water and frozen at −20 °C, and were then used as stock raw material throughout the study.

### 2.2. Biological Materials and Chemicals

The yeast strain *C. rhodanensis* DK used in this research was isolated from a sample of laphet-so, a fermented tea collected from the southern Shan state of Myanmar [18]. *Saccharomyces cerevisiae* TISTR 5088, obtained from the culture collection of the Thailand Institute of Scientific and Technological Research (TISTR), and *Lactobacillus pentosus* A14-6, tannin-tolerant with probiotic properties originally isolated from samples of miang [21], were used in the study. All yeast and bacterial strains were maintained as stock cultures in 50% (*v*/*v*) glycerol and stored at −20 °C, for further use in co-fermentation to produce fermented cashew beverages. The yeast cultures were sustained in yeast malt extract (YM) media throughout the experimental procedures. The bacterial strain was maintained in de Man, Rogosa, and Sharpe (MRS) media at 4 °C for short-term storage and in a broth supplemented with 50% (*v*/*v*) glycerol at −20 °C for long-term storage. All chemicals used for preparing solvents, reagents, and buffers were of analytical grade. The analytical grade of *p*-nitrophenyl-α-D-glucopyranoside, *p*-nitrophenyl-β-D-glucopyranoside, and the purified α-glucosidase from *Saccharomyces cerevisiae* were purchased from Sigma-Aldrich (St. Louis, MO, USA).

### 2.3. Microbial Growth and Ethanol Production of C. rhodanensis DK in Various Glucose Concentrations

The capability of growth and ethanol fermentation of *C. rhodanensis* DK was investigated in a 2 L Erlenmeyer flask containing 1000 mL of yeast malt extract (YM) broth with varied glucose concentrations ranging from 0 to 20% (*w*/*v*). After sterilization through autoclaving at 121 °C for 15 min, each YM broth was inoculated with seed inoculum of *C. rhodanensis* DK at 10% (*v*/*v*) (6.54 ± 0.06 Log cfu/mL), and statically cultivated at 30 °C for 12 days. The culture broth was sampled every day for assessment of microbial growth and ethanol production. The pH values were measured using a pH meter, and the viable yeast cell was enumerated by the drop plate technique after a 10-fold serial dilution using a 0.85% (*w*/*v*) NaCl solution [14]. The reducing sugar was analyzed by the DNS method [22], using glucose as a standard sugar. Additionally, ethanol contents were measured by high-performance liquid chromatography (HPLC) as described below.

### 2.4. Evaluation of Microbial Growth and Ethanol Productivity of C. rhodanensis DK under Different Compositions of Cashew Apple Juice

The cashew apple waste was cut into small cubic pieces (approximately 2 cm^3^) and homogenized using a Masticator blender (IUL, SA, Barcelona, Spain) for 5 min. The homogenized cashew apple juice was filtered through a stainless-steel sieve mesh (150 µm, 400 mm in diameter, 50 mm depth). The aqueous extract produced after filtration was defined as cashew apple juice (CAJ). The CAJ had an initial pH value of 4.6 and a sugar concentration of 6.8% (*w*/*v*).

CAJs (1000 mL) with different sugar content and pH were prepared as follows: (1) pure CAJ with the original pH of 4.6 and 6.8% sugars, (2) CAJ with pH adjusted to 6 and 6.8% sugars, (3) CAJ with the original pH of 4.5 and sugars adjusted to 5% (*w*/*v*), and (4) CAJ with the original pH of 4.5 sugars adjusted to 10% (*w*/*v*). The preparation of the different CAJ formulations involved specific methods. To achieve a pH of 6, approximately 6 mL of 10 M NaOH was added to pure CAJ while maintaining the sugar concentration at 6.8% (*w*/*v*). Furthermore, CAJ was diluted with distilled water to attain 5% (*w*/*v*) sugar concentration; this dilution resulted in a starting pH of 4.5 for this concentration. Adding glucose to CAJ to achieve a final concentration of 10% (*w*/*v*) glucose did not affect the final pH, which remained at 4.5 after adding glucose. After autoclaving the CAJs for 15 min at 121 °C, each sample was inoculated with 10% (*v*/*v*) (6.57 ± 0.03 Log cfu/mL) of *C. rhodanensis* DK seed culture, and the fermentation process was carried out at 30 °C under static conditions for 12 days. Sampling of the culture broth was carried out daily throughout the fermentation process to monitor the pH and conduct cell viability counts. The sugars and ethanol concentration in the cell-free supernatant were determined by HPLC following the methodology and conditions described by Kodchasee et al. [14]. Sugar consumption and ethanol production were then calculated based on the initial sugar concentration.

### 2.5. Co-Culture Fermentation of Cashew Apple Juice by C. rhodanensis DK with S. cerevisiae TISTR 5088 or L. pentosus A14-6

CAJ was prepared as described previously, and 1 L of CAJ was transferred into a 2 L Erlenmeyer flask with pH adjusted to 6 before sterilization by autoclaving at 121 °C for 15 min. Microbial inoculums were prepared by inoculation of a single colony of yeast and lactic acid bacterial (LAB) strains in yeast malt extract (YM) and de Man, Rogosa, and Sharpe (MRS) broth, respectively, and the cultures were incubated at 30 °C. Yeasts were incubated for 48 h, while the LAB strains were incubated for 24 h. Inoculums (10% (*v*/*v*)) of each microbe were transferred into sterile CAJ, and the fermentation process was performed at 30 °C for 7 days. A total of four treatments consisted of (1) control group: CAJ without inoculation, (2) CAJ with 10% (*v*/*v*) (6.64 ± 0.03 Log cfu/mL) *C. rhodanensis* DK inoculum, (3) CAJ with 5% (*v*/*v*) (6.23 ± 0.01 Log cfu/mL) *C. rhodanensis* DK and 5% (*v*/*v*) (6.34 ± 0.02 Log cfu/mL) *S. cerevisiae* TISTR 5088 inoculums, and (4) CAJ with 5% (*v*/*v*) (6.23 ± 0.01 Log cfu/mL) *C. rhodanensis* DK and 5% (*v*/*v*) (7.01 ± 0.01 Log cfu/mL) *L. pentosus* A14-6 inoculums. Treatments 3 and 4 were initially started with 5% (*v*/*v*) *C. rhodanensis* DK on day 0, and the co-cultures in treatments 3 and 4 were initiated after 3 days of fermentation until day 7.

### 2.6. Analysis of Biological and Physicochemical Properties of the Fermented Products

#### 2.6.1. Viable Cell Counts

Viable cell counts were determined by the drop plate technique as described previously. Briefly, 0.1 mL suspension of the appropriately diluted sample was then triplicated spread onto yeast malt extract (YM) agar for yeasts and de Man, Rogosa, and Sharpe (MRS) agar for LAB. Subsequently, the agar plate cultures were incubated at 30 °C for 24 to 48 h, and a single colony of each microbe was enumerated and presented as colony forming units (cfu) in terms of Log cfu/mL.

#### 2.6.2. pH and Total Titratable Acidity

Throughout the fermentation periods, the pH values of the fermented CAJ samples were monitored using a pH meter. To determine the total titratable acidity (TTA), each sample was titrated with 0.01 M NaOH solution using phenolphthalein as an indicator following the method described by AOAC [23]. The TTA was calculated following the AOAC standard protocol and expressed in molar (M).

#### 2.6.3. Total Sugar and Reducing Sugar

Total sugar contents were analyzed as glucose equivalents by the phenol sulfuric acid method of DuBois et al. [24]. Reducing sugar contents were analyzed as glucose equivalents by the DNS method [22]. Absorbance was recorded using a spectrophotometer (Metertech SP-8001 UV/Visible Spectrophotometer, Metertech Inc., Taipei, Taiwan).

#### 2.6.4. Sugars and Ethanol

The quantification of sugars (glucose, sucrose, and fructose) and ethanol was conducted using HPLC following the method described by Kodchasee et al. [14]. Briefly, 10 µL aliquots of the filtered samples were injected into an HPLC system (HPLC: HITACHI) equipped with a dual detection setup comprising a UV detector (DAD) and a refractive index detector (RID). The HPLC analysis was performed using an Aminex HPX-87H, 300 × 7.8 mm column (Bio-Rad, Hercules, CA, USA), with 5 mM of H_2_SO_4_ as the mobile phase, a flow rate of 0.75 mL/min, and a column compartment temperature of 40 °C. The run time was set at 20 min, with an operating temperature of 65 °C. All samples were analyzed in triplicates; standard solutions were also injected to determine the retention times for each compound, and glycerol (*v*/*v*) was used as the internal standard.

### 2.7. Analysis of Bioactive Compounds and Antioxidant Activity

#### 2.7.1. Total Polyphenols

The total polyphenol (TP) content was determined following the Folin-Denis method, as described by Abdullahi et al. [25]. In brief, 250 µL of the sample was diluted with 1625 µL of deionized (DI) water, resulting in a total volume of 1875 µL. Then, 125 µL of Folin-Denis reagent (2M) was added to the sample mixture and mixed thoroughly by vortexing (Scientific Industries, Bohemia, NY, USA). Subsequently, 250 µL of sodium carbonate (10%, *w*/*v*) was added and mixed by vortexing. The final sample volume was adjusted to 2.5 mL by adding 250 µL of DI water, and the absorbance of the final mixture was measured at 750 nm. DI water served as the blank, while gallic acid (GA) was used as the standard for comparison. The results of total polyphenols are expressed in mg of GA equivalents per gram of the sample (GAE/g).

#### 2.7.2. Total Tannins

The total tannin (TT) content was determined using the Folin-Denis method with the addition of polyvinylpolypyrrolidone (PVPP) to isolate tannins from other phenols, following the procedure described by Makkar et al. [26]. Briefly, 1 mL of the sample solution was mixed with 1 mL of PVPP (10% *v*/*v*) and vortexed, then left at 4 °C for 15 min. Afterward, the mixture was centrifuged at 3000 rpm for 10 min, and the resulting supernatant was collected. The supernatant was then assessed using the Folin-Denis reagent, and the TT content was calculated using the formula: TT = TP − PVPP precipitated supernatant, where TP represents total polyphenols. The absorbance of the final mixture of all samples was measured at 750 nm. The results are expressed as mg of tannic acid equivalents per gram of the sample (TAE/g).

#### 2.7.3. Total Flavonoids

The total flavonoid (TF) content was determined using the aluminum chloride colorimetric method. In brief, 250 µL of the sample was mixed with 50 µL of 10% (*w*/*v*) of aluminum nitrate and 50 µL of 1M potassium acetate. The mixture was then adjusted to a total volume of 2 mL by adding 1650 µL of 80% methanol, followed by vigorous vortexing, and incubated at room temperature (20–30 °C) for 40 min. The absorbance of all samples was measured at 415 nm. For the blank control, 80% methanol was used, and quercetin (QE) was used as the standard for comparison. The results of the TF content are expressed in mg of QE equivalents per gram of the sample (QE/g) [27].

#### 2.7.4. Antioxidant Activity

The antioxidant capacity of the samples was assessed using the DPPH (2,2-difenil-1-picrilhidrazyl hydrate) (Sigma-Aldrich, St, Louis, MO, USA) free radical assay, as outlined by Unban et al. [21]. In this method, 100 µL of the sample was mixed with 400 µL of the DPPH solution, which had been prepared at a concentration of 0.15 mM with 80% of methanol. The mixtures were thoroughly shaken and then left at room temperature in the dark for 30 min. Following the incubation, the absorbance of the samples was measured by recording the decrease in absorbance at 517 nm. The percentage antioxidant potential of the samples was calculated using a specific equation. The percentage potential was estimated using the following equation:

DPPH scavenging capacity (%) = (A_control_ − A_sample_)/A_control_ × 100 (1)

where A is absorbance.

### 2.8. β-Glucosidase Assay

The enzyme activity of β-glucosidase in the fermented products was determined following the method described by Sørensen et al. [28]. A concentration of 4.0 mM of *p*NPG was used as a substrate for β-glucosidase. Briefly, 50 µL of enzyme supernatant was mixed with 50 µL of 4.0 mM *p*NPG to assay for β-glucosidase activity. The mixture was independently incubated at 37 °C for 30 min. The enzyme reaction was halted by introducing 100 µL of 50 mM Na_2_CO_3_. Subsequently, the absorbance of the final reaction mixture was measured at 405 nm by a spectrophotometer (Metertech SP-8001 UV/Visible Spectrophotometer, Metertech Inc., Taipei, Taiwan). A blank solution containing 100 mM of citrate phosphate buffer (pH 6) was used as a reference for the substrate and enzyme blanks as well.

### 2.9. Determination of In Vitro α-Glucosidase Inhibitory Activity

The preliminary experiment for determining the deactivation of α-glucosidase in the fermented CAJ samples was carried out by incubation at a temperature ranging from 30 to 80 °C [29], and the completely deactivated α-glucosidase condition was selected for further experiment. Subsequently, the α-glucosidase inhibitory activity of the α-glucosidase-eliminated samples was determined following a method described by Kaprasob et al. [4]. Briefly, 50 µL of the sample was mixed with 50 µL of 1.0 M phosphate buffer. Then, 100 µL of α-glucosidase enzyme solution (1.0 U/mL, prepared in phosphate buffer pH 6.9) was added into the mixture containing the sample, followed by incubation for 10 min at 30 °C. Following this pre-incubation period, 50 µL of substrate (5.0 mM *p*-nitrophenyl-α-glucopyranoside solution, prepared in the same phosphate buffer as the enzyme solution) was added and continuously incubated for 5 min. The reaction was stopped by adding 300 µL of 0.1 M Na_2_CO_3_. The absorbance of the reaction mixture was measured at 405 nm by a spectrophotometer. The absorbance of the sample containing 50 µL of potassium phosphate buffer (pH 6.9) instead of the sample was used as the control. The relative α-glucosidase inhibitory activity (%) was calculated by the following equation:

% inhibition = (A_control_ − A_sample_)/A_control_ × 100 (2)

where A is absorbance.

### 2.10. Statistical Analysis

The statistical values, including the mean ± standard deviation (SD), were derived from two separate and independent trials. These values were obtained using a one-way analysis of variance (ANOVA) followed by a post hoc test (Tukey) using the IBM Statistics SPSS software 20. The significance level for all analyses was set at *p* < 0.05.

## 3. Results

### 3.1. Microbial Growth and Ethanol Production of C. rhodanensis DK in Various Glucose Concentrations

**Figure 1:**
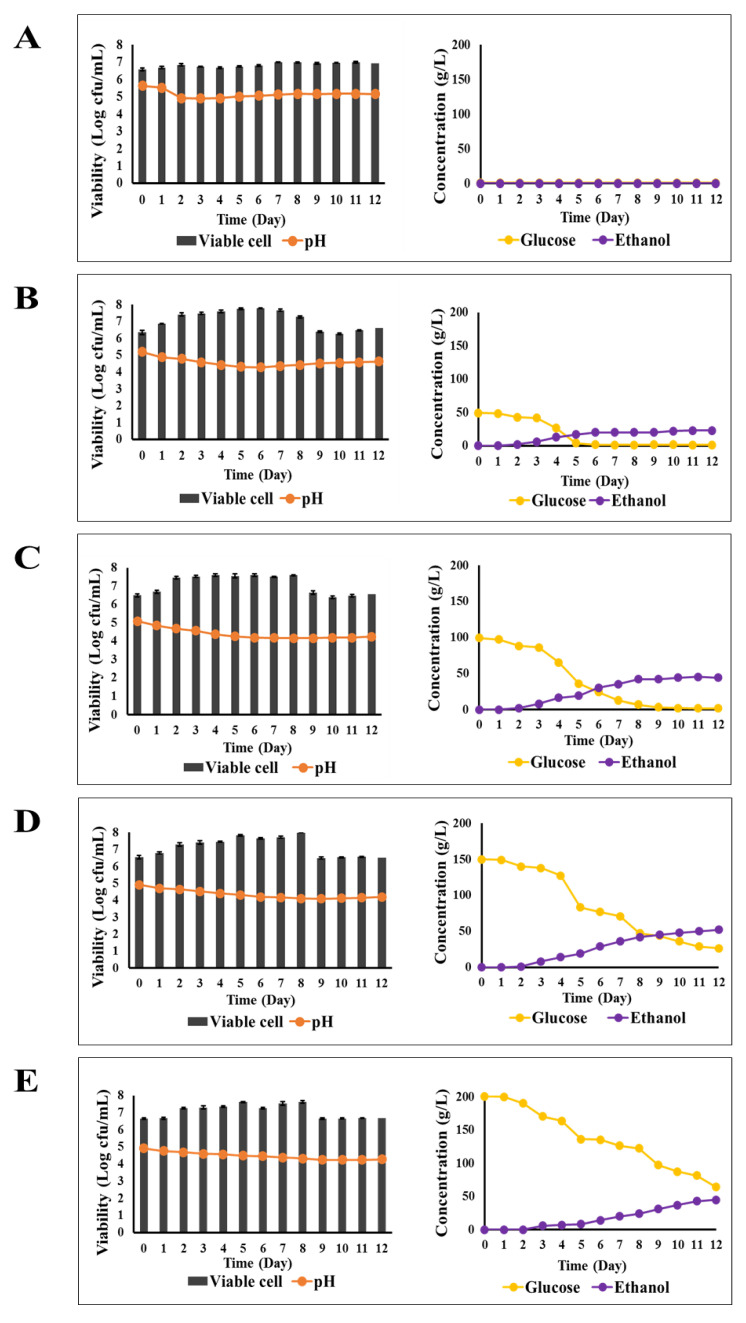
Changes in pH, viable cell growth, glucose, and ethanol during 12 days of fermentation by *C. rhodanensis* DK at 30 °C in YM broth with different glucose concentrations as a carbon source: (**A**) 0%, (**B**) 5%, (**C**) 10%, (**D**) 15%, and (**E**) 20%. Data expressed as mean ± SD (*N* = 3).

The changes in pH, viable cells of *C. rhodanensis* DK, sugar consumption, and ethanol production for 12 days of fermentation in YM broths containing different sugar concentrations are presented in Figure 1. The result of 0% glucose (control) showed that the pH and the viability of yeast cells were rather stable throughout the entire fermentation period. There was no detection of sugar consumption and ethanol production during 12 days of fermentation. The culture with 5% (*w*/*v*) glucose revealed a decrease in pH from 5.19 to 4.62 after fermentation, while the viable cells also increased by approximately 1 Log cfu/mL. All the sugar was consumed by yeast, and ethanol accumulation in the culture broth was 21 g/L (2.1%) after fermentation. At a level of 10% glucose concentration, the pH gradually decreased to 4.18, and the viable cells increased from 6.49 to 7.50 Log cfu/mL during fermentation on day 8 and gradually decreased after fermentation. The yeast consumed all the sugar from 10% (*w*/*v*) glucose to the remaining 0.1% (*w*/*v*), and ethanol production was detected up to 4.5% at the end of the fermentation process. In the case of 15% (*w*/*v*) and 20% (*w*/*v*) glucose concentrations, the pH gradually decreased to the final pH of 4.19 and 4.25, respectively, while the patterns of viable cell growth rates of both concentrations were similar to that of 10% (*w*/*v*) glucose concentration. The results showed that *C. rhodanesis* DK could not consume all of the sugar in both glucose concentrations, and the ethanol concentrations at 12 days fermentation of 15% (*w*/*v*) and 20% (*w*/*v*) initial glucose were 5.2% and 4.5%, respectively. These results indicate the influencing effect of high sugar concentration on ethanol production capability during fermentation. Gemilang et al. [30] reported that sugar concentration played an active role in influencing the rate of ethanol generation. Our investigation showed that 5 and 10% glucose concentration resulted in complete fermentation within 12 days. These results were similar to those of Chang et al. [31], who reported that at an initial glucose concentration of 4%, the glucose was depleted within a short time, and sugar consumption rates were more delayed at glucose concentrations up to 10% and exhausted for supporting ethanol production. Furthermore, the results of our study revealed that the use of sugar concentrations of 15 and 20% in single fermentation is less efficient in terms of both sugar consumption and ethanol production. These results are aligned with the findings of Lee & Park [32], who mentioned that up to 20% of glucose concentration results in osmotic stress on non-*Saccharomyces* yeast in the production of Lorean Muscat Bailay wine. Furthermore, da Cruz et al. [33] also mentioned that sugar concentration had a strong effect on the shift from reductive fermentative to oxidative metabolism. Collí et al. [34] reported that when the sugar concentration in the medium increases, it creates a gradient of osmotic pressure, because the higher osmotic pressure leads to water loss through osmosis, causing a decrease in cell volume and limiting the amount of water available for essential cellular processes.

### 3.2. Evaluation of Microbial Growth and Ethanol Productivity of C. rhodanensis DK under Different Compositions of Cashew Apple Juice

**Figure 2:**
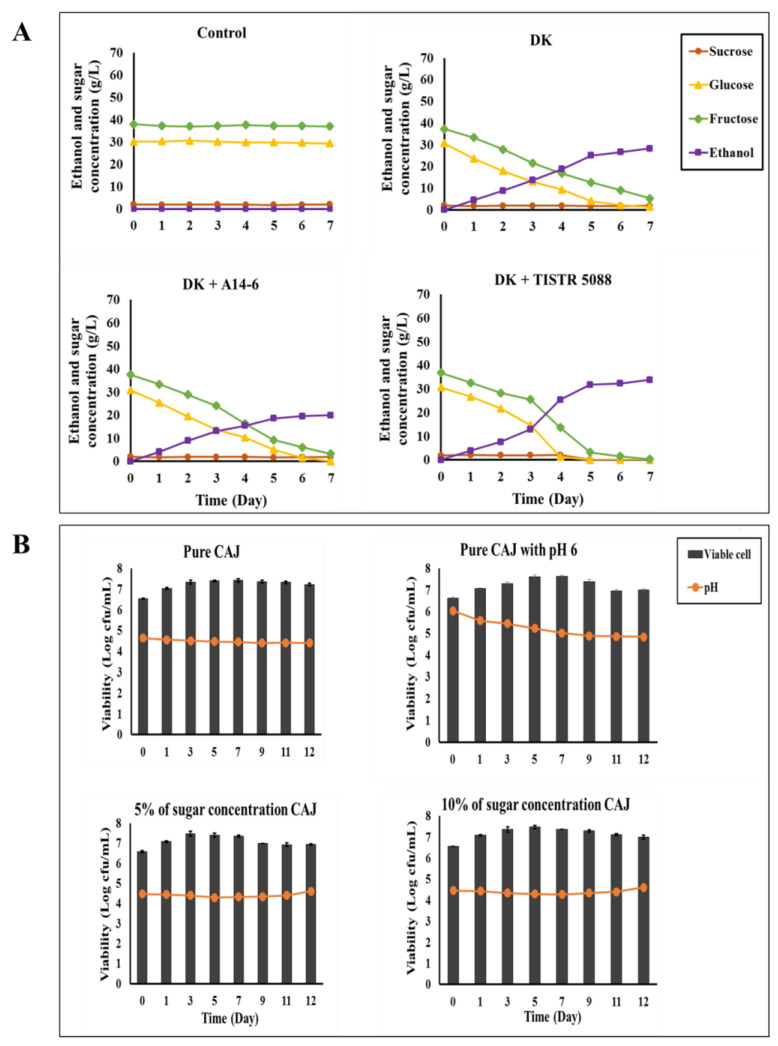
Changes in sugars and ethanol (**A**), cell viability, and pH (**B**) during 12 days of fermentation by *C. rhodanensis* DK in various conditions of cashew apple juice (CAJ) at 30 °C. Data expressed as mean ± SD (*N* = 3).

This study examined the influences of four different compositions and properties of CAJ on the microbial growth and ethanol productivity of *C. rhodanensis* DK: (1) original CAJ, (2) CAJ with pH 6 and 6.8% (*w*/*v*) sugars, (3) CAJ with 5% sugar concentration, and (4) CAJ with 10% sugar concentration. This preliminary investigation focused on assessing changes in pH, viable cell count, consumption of sugars, and ethanol production capability to identify the optimal CAJ composition for further experimentation. Our findings indicated that the initial CAJ used in this study contained mainly glucose (31.01 ± 0.33 g/L), fructose (37.31 ± 0.02 g/L), and a minor presence of sucrose (1.55 ± 0.02 g/L). This study outcome aligns with the findings of Gamero et al. [5], who reported the predominant sugar content in CAJ. In this study, the results from fermenting pure CAJ showed the complete consumption of glucose, while approximately 8.54 ± 0.24 g/L of fructose remained. Ethanol production reached 27.06 ± 0.16 g/L, accompanied by a slight decrease in pH to 4.41 and viable cell count increased to 7.22 ± 0.06 Log cfu/mL over the 12-day fermentation period. Following this, when studying pure CAJ with a pH adjusted to 6, the results indicated that both glucose and fructose in the juice were completely consumed. This led to higher production of ethanol at a level of 31.52 ± 0.02 g/L, a decrease in pH to 4.84 after 12 days of fermentation, and an increase in viable cell count to 7.08 ± 0.03 Log cfu/mL after 1 day. Notably, this result demonstrated the complete utilization of sugars in CAJ and a significant increase in ethanol production compared to the composition of pure CAJ. In CAJ with the addition of a 5% sugar concentration, the fermentation process led to a decrease in pH to 4.61, and the viable cell count increased to 7.39 ± 0.07 Log cfu/mL. Complete consumption of glucose was observed, and approximately 0.77 ± 0.23 g/L of fructose remained, while ethanol production reached 23.65 ± 0.05 g/L after 12 days fermentation period. Furthermore, in the analysis of a 10% sugar concentration in CAJ, complete glucose consumption was observed, and fructose levels were approximately 7.56 ± 0.23 g/L at the end of the fermentation period. Ethanol production reached 39.82 ± 0.24 g/L, while the pH changed to 4.61. The viable cell count remained at approximately 7.02 ± 0.04 Log cfu/mL throughout the fermentation process. Sucrose concentration remained constant across all CAJ compositions throughout the whole experiment, as shown in Figure 2.

Our study showed that CAJ is rich in reducing sugars (glucose and fructose), with a pH of 4.41–4.64. Moreover, the observed changes in pH, viable cell count, sugar reduction, and ethanol production after fermentation provide valuable insights into the capability of *C. rhodanensis* DK to adapt under different CAJ conditions. Notably, CAJ compositions such as pure CAJ adjusted to pH 6 and 5% sugar concentration demonstrated complete fermentation within 12 days. In contrast, incomplete fermentation in pure CAJ pH 4.41 with 10% sugar concentration may be attributed to non-*Saccharomyces* yeasts facing challenges in fully consuming the available nutrients in fruit juice during fermentation processes. This observation aligns with the results of Gschaedler et al. [35]. The complete fermentation in pure CAJ with pH adjusted to 6 at 5% sugar concentration was suggested to be a potential pH effect on yeast glycolysis during fermentation. Mohd-Zaki et al. [36] reported that pH of the environment has a significant impact on the efficiency of glycolysis and ethanol production. The ethanol yields at pH values outside the optimal pH range of enzyme activities involved in glycolysis and ethanol production can be significantly reduced. Therefore, monitoring and adjusting pH levels during ethanol production are crucial for ensuring optimal yeast growth and fermentation efficiency.

### 3.3. Analysis of Biological and Physicochemical Properties of the Fermented Products

#### 3.3.1. Viable Cell Counts

**Figure 3:**
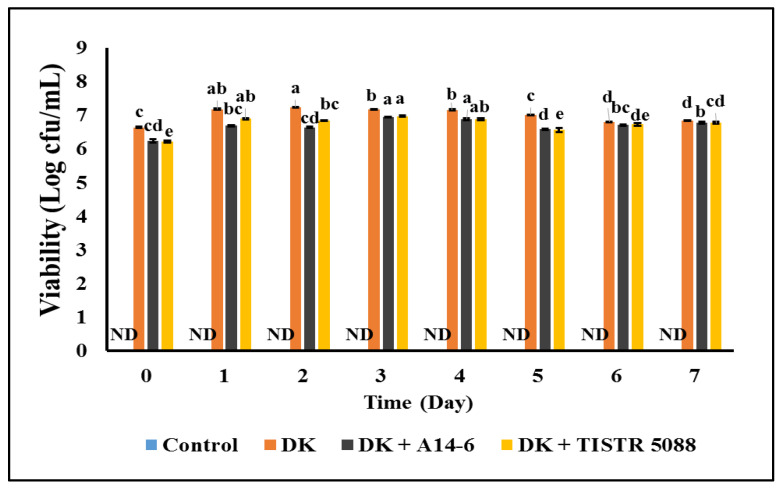
Changes in viable cells during fermentation at 30 °C for seven days by *C. rhodanensis* DK (DK); co-culture of *C. rhodanensis* DK + *L. pentosus* A14-6 (DK + A14-6); co-culture of *C. rhodanensis* DK + *S. cerevisiae* TISTR 5088 (DK + TISTR 5088); and non-inoculated (Control). Different letters (a–e) indicate significant differences in the values (*p* < 0.05). Data are expressed as mean ± SD (*N* = 3). ND represents no viable cells were detected.

The changes in yeast population dynamics during mono- and co-fermentation of CAJs are described in Figure 3. In mono-culture fermentation, the viable cell count of *C. rhodanensis* DK exhibited a gradual increase from 6.64 ± 0.03 to 7.23 ± 0.02 Log cfu/mL within three days, followed by a subsequent decline to 6.83 ± 0.02 Log cfu/mL over the fermentation period. During the early stages of co-culture fermentation, the viable cell count of *C. rhodanensis* DK increased from 6.23 ± 0.01 to 6.95 ± 0.01 Log cfu/mL on day 3 of fermentation. Furthermore, co-culture fermentation involving *C. rhodanensis* DK + *S. cerevisiae* TISTR 5088 showed changes in cell growth, ranging from 6.21 ± 0.02 to 6.98 ± 0.02 Log cfu/mL, later gradually decreasing to 6.77 ± 0.03 Log cfu/mL during fermentation. Similarly, in the co-culture of *C. rhodanensis* DK+ *L. pentosus* A14-6, the yeast population increased in the first three days of fermentation; however, after the addition of *L. pentosus* A14-6, the yeast population gradually decreased to 6.77 ± 0.03 Log cfu/mL.

The results showed that the yeast populations gradually increased in both mono- and co-culture fermentations during the first two days and later gradually decreased over the fermentation periods. This trend aligns with the findings of Sudun et al. [37], who observed an initial increase in yeast growth within the first two days, followed by a gradual decline after seven days of fermentation. The cell population increase during the first two days could be that the cells obtained sufficient nutrients, and the gradual decrease in the cell population may be attributed to oxidative or weakly fermentative metabolism, coinciding with the depletion of available sugars or the effect of ethanol concentration [38]. Furthermore, the yeast population after the addition of yeast *S. cerevisiae* TISTR 5088 showed insignificant changes. Kim et al. [39] mentioned that when there is no significant change in a cell population, it may not significantly influence the cell growth and ethanol concentration of yeast cells. Additionally, the gradual decrease in yeast population after the addition of *L. pentosus* A14-6 may be due to oxidative or weakly fermentative metabolism, or LAB addition leads to an increase in acidity during the later stages of fermentation, as the optimal survival pH range for yeasts is typically between 5 and 6. This result is consistent with the findings reported by Cai et al. [40], who suggested that the decline in yeast population after the addition of LAB may be associated with increased acidity. These findings highlight the dynamic nature of yeast interactions in different fermentation conditions in monoculture and co-culture fermentations.

#### 3.3.2. pH and Total Titratable Acidity

**Figure 4:**
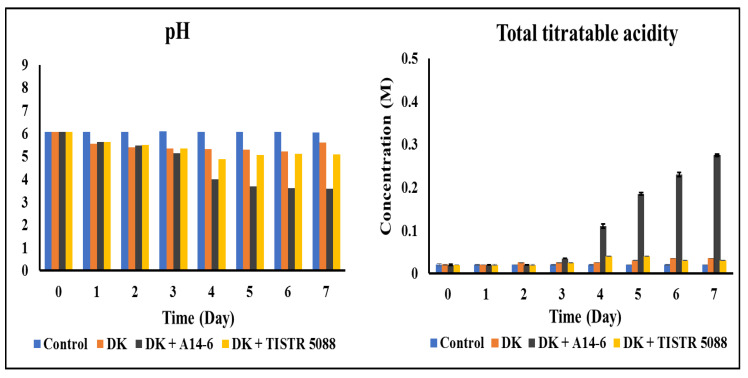
Changes in pH and total titratable acidity of fermented CAJ during fermentation at 30 °C for seven days by *C. rhodanensis* DK (DK), *C. rhodanensis* DK + *L. pentosus* A14-6 (DK + A14-6), *C. rhodanensis* DK + *S. cerevisiae* TISTR 5088 (DK + TISTR 5088), and non-inoculated (Control). Data expressed as mean ± SD (*N* = 3).

The variations in pH and total titratable acidity (TTA) during the fermentation process of CAJ are demonstrated in Figure 4. Initially, the pH of control CAJ was 6.05, with a low TTA of 0.02 ± 0.01 M. Following fermentation, CAJ fermented with *C. rhodanensis* DK or mono-fermentation showed a gradual decrease in pH from 6.05 to 5.61, accompanied by a slight increase in TTA from 0.02 ± 0.01 to 0.04 ± 0.01 M. In co-culture fermentation with *C. rhodanensis* DK + *S. cerevisiae* TISTR 5088, the pH decreased to 5.09, and total acidity increased from 0.02 ± 0.01 to 0.03 ± 0.01 M after seven days of fermentation. Notably, co-culture fermentation with *C. rhodanensis* DK + *L. pentosus* A14-6 resulted in a significant decrease in pH to 3.58, accompanied by a substantial increase in TTA to 0.28 ± 0.01 M after fermentation. Our study demonstrates distinct variations in the pH and total acidity of fermented CAJ with mono- and co-culture fermentation in comparison to the control, emphasizing the distinct influence of specific inoculums on the fermentation process. According to the results, the utilization of yeasts for fermentation led to a gradual decrease in pH and an increase in total acidity of CAJ over the fermentation period. This slight pH decline and increase in TTA may be attributed to the production of organic acids by yeasts [41]. In the case of co-culture fermentation with *L. pentosus* A14-6, a sharp decrease in pH and an increase in total acidity compared to other yeast inoculums were observed. This pH reduction and total acidity increase can be attributed to the lactic acid production by lactic acid bacteria (LAB), such as *L. pentosus* A14-6. This observation aligns with the finding of Mousavi et al. [42], who highlighted the pH-lowering and acidity-increasing capabilities of probiotic lactic acid bacteria during fermentation. Similarly, Kaprasob et al. [4] documented a comparable occurrence in cashew apple fermentation, where LAB played a role in pH reduction due to its production of organic acids. This suggests that the presence of *L. pentosus* A14-6 in the co-culture significantly influenced the acidification of the CAJ, potentially impacting its flavor profile. Our findings underscore the impact of different inoculums on the pH and total acidity of fermented CAJ, providing valuable insights into the potential alterations in flavor and quality induced by specific microbial interactions during the fermentation process. This distinct effect is attributed to the ability of *L. pentosus* to produce lactic acid, further acidifying the CAJ [42]. This acidification may impact the flavor profile and potentially influence the health benefits of fermented CAJ due to the organic acid produced by yeast and LAB, which possess antimicrobial properties [43].

#### 3.3.3. Total Sugar and Reducing Sugar

**Figure 5:**
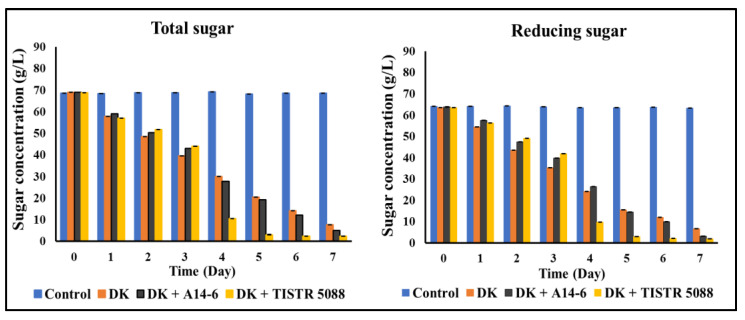
Changes in total sugar and reducing sugar of fermented CAJ inoculated with non-inoculated (Control), *C. rhodanensis* DK (DK), *C. rhodanensis* DK + *L. pentosus* A14-6 (DK + A14-6), and *C. rhodanensis* DK + *S. cerevisiae* TISTR 5088 (DK + TISTR 5088) during CAJ fermentation at 30 °C for seven days. Data expressed as mean ± SD (*N* = 3).

Changes in total sugar and reducing sugar contents throughout the fermentation of CAJ with various microbial inoculums are shown in Figure 5. Initially, CAJ (control) had total sugar at 68.52 ± 0.01 g/L and reducing sugar at 64.05 ± 0.02 g/L. After seven days of fermentation, mono-culture fermentation with *C. rhodanensis* DK resulted in the total and reducing sugar levels of fermented CAJ remaining relatively stable at 7.70 ± 0.01 g/L and 6.70 ± 0.03 g/L, respectively. In contrast, co-culture fermentation with *C. rhodanensis* DK + *S. cerevisiae* TISTR 5088 led to a sharp decrease in both total sugar and reducing sugar contents. At the end of the fermentation process, total sugar content dropped to 2.33 ± 0.02 g/L, and reducing sugar content reached 1.82 ± 0.01 g/L. Similarly, co-culture fermentation involving *C. rhodanensis* DK + *L. pentosus* A14-6 resulted in a total sugar content of 4.96 ± 0.02 g/L, while the reducing sugar was 3.04 ± 0.03 g/L after seven days of fermentation.

The observed variations in total sugar and reducing sugar contents provide valuable insights into sugar metabolism during the fermentation of CAJ with different microbial inoculums. When CAJ undergoes fermentation with *C. rhodanensis* DK as a monoculture, levels of both total and reducing sugar remain at 7.71 ± 0.01 g/L and 6.70 ± 0.03 g/L, respectively. This could be attributed to the metabolic preferences of *C. rhodanensis* DK, which may not be highly efficient at metabolizing the sugars present in CAJ. This aligns with the common behavior of non-*Saccharomyces* yeasts, leaving residual sugars [44]. In contrast, co-culture fermentations with *C. rhodanensis* DK + *S. cerevisiae* TISTR 5088 and *C. rhodanensis* DK + *L. pentosus* A14-6 demonstrated enhanced efficiency in utilizing available sugars. The drastic decrease in sugar levels suggests that co-culture fermentation is efficient in sugar fermentation in CAJ. The decrease in sugar levels can likely be attributed to the metabolic activities of *S. cerevisiae* TISTR 5088 and *L. pentosus* A14-6 strains, which are renowned for their efficiency in fermenting sugars [45,46]. Notably, the presence of *S. cerevisiae* in co-culture fermentation is associated with complete sugar consumption, leading to less sweet final products. Similarly, *L. pentosus* strains contribute to sugar utilization and acid production during fermentation, influencing the sugar content of the final product.

#### 3.3.4. Sugars and Ethanol

**Figure 6:**
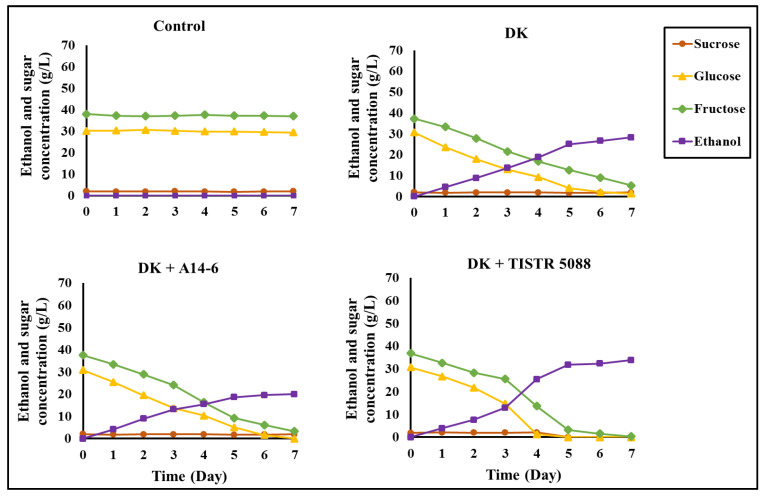
Changes in ethanol and sugar concentrations during fermentation of non-inoculated (Control) CAJ, and CAJ inoculated with *C. rhodanensis* DK (DK), *C. rhodanensis* DK + *L. pentosus* A14-6 (DK + A14-6), and *C. rhodanensis* DK + *S. cerevisiae* TISTR 5088 (DK + TISTR 5088) at 30 °C for seven days.

In the CAJ (control), glucose and fructose were present at concentrations of 30.13 ± 0.14 g/L and 37.49 ± 0.15 g/L, respectively, with an additional 2.13 ± 0.03 g/L of sucrose. The ethanol and sugar contents of low-alcohol beverages were derived from CAJ, highlighting variations in sugar conversion during fermentation as presented in Figure 6. During mono-fermentation with *C. rhodanensis* DK, ethanol production was initiated within 24 h and reached a maximum of 28.23 ± 0.16 g/L after seven days. The remaining sugar content consisted of 1.39 ± 0.04 g/L of glucose and 5.21 ± 0.16 g/L of fructose, with a constant sucrose content after seven days of fermentation. In co-culture fermentation with *C. rhodanensis* DK + *S. cerevisiae* TISTR 5088, the fermentation rate accelerated, resulting in ethanol production of 33.61 ± 0.11 g/L, with complete glucose consumption and residual fructose content of 0.24 ± 0.03 g/L after seven days of fermentation. Furthermore, co-fermentation with *C. rhodanensis* DK + *L. pentosus* A14.6 yielded a final ethanol production of 19.47 ± 0.06 g/L. All glucose was consumed, leaving a residual fructose content of 3.20 ± 0.04 g/L, with no change in sucrose content at the end of the fermentation.

These findings highlight the significant impact of different microbial strains on the sugar conversion of specific combinations of strains. In mono-culture fermentation with *C. rhodanensis* DK, sugar levels remain, indicating the potential inefficiency in metabolizing CAJ sugars fully, while *S. cerevisiae* TISTR 5088 emerges as a promising candidate due to its efficient and rapid sugar conversion capabilities, contributing to enhanced ethanol production [47]; moreover, the utilization of *L. pentosus* A14.6 in CAJ fermentation presents a distinctive dynamic. Although *L. pentosus* A14.6 actively participates in sugar consumption during fermentation, its acid-producing characteristic contributes to the retention of residual sugars and a subsequent decrease in ethanol production. This observation aligns with the finding of Cai et al. [40], who highlighted the essential role of lactic acid bacteria in facilitating efficient glucose consumption, resulting in lower pH and ethanol volume. Consistent with Kaprasob et al. [4], our study observed a correlation between the remaining sugar contents, a decrease in pH, and an increase in total acidity. These interconnected interactions emphasize the intricate dynamics of the fermentation process, where changes in pH and acidity influence the fermentation environment, subsequently affecting microbial activities, sugar consumption, and product outcomes. Additionally, the influence of pH on the production of microbial metabolites during glucose feeding is well-documented, especially in the availability of protons, governed by pH levels, playing a role in influencing reductase activity. This, in turn, has far-reaching effects on both intracellular and extracellular microbial activities, as demonstrated in prior research by Mohd-Zaki et al. [36]. The correlation between sugar utilization and pH changes supports the intricate dynamics of fermentation. This study investigated the changing profiles of sugars and ethanol during fermentation using both mono- and co-culture approaches, with the depletion of sugars in fermented CAJ having the potential to provide health benefits. Understanding these relationships is vital for optimizing fermentation processes, enhancing ethanol production, and maintaining the quality of low-alcohol beverages derived from cashew apple juice for health benefits.

### 3.4. Analysis of Bioactive Compounds and Antioxidant Activity

#### 3.4.1. Total Polyphenols

**Figure 7:**
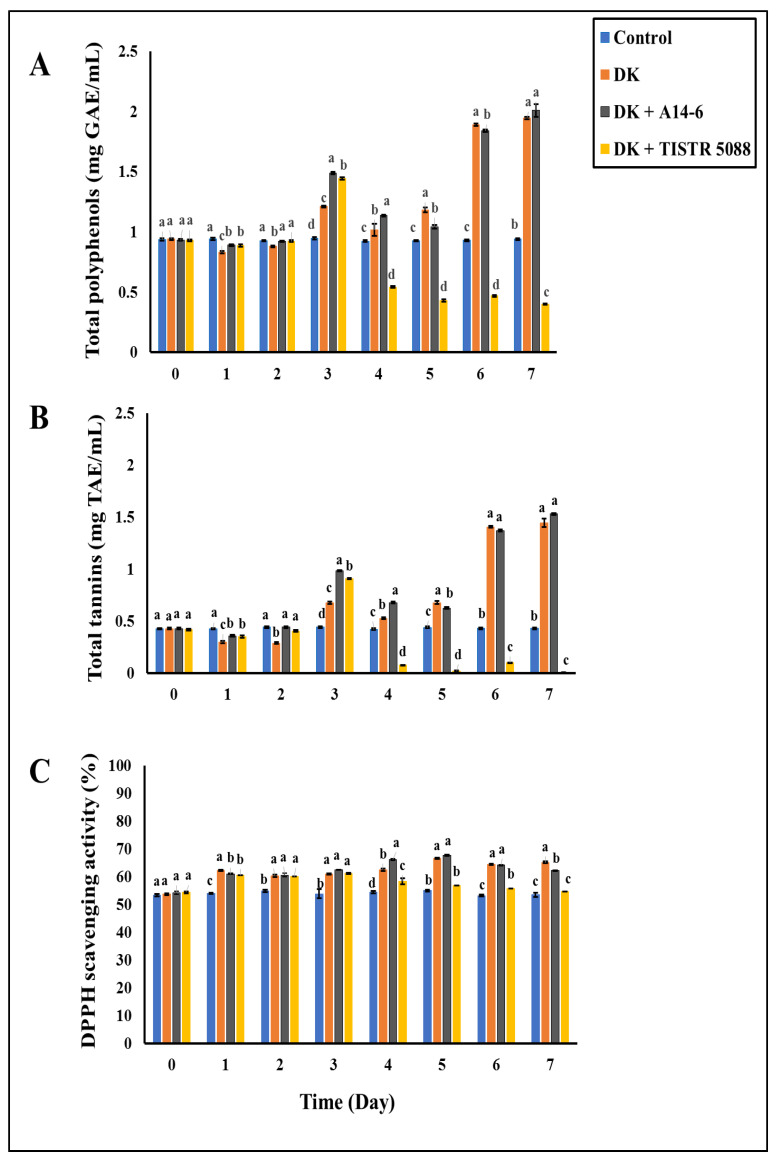
Changes in total polyphenols (**A**), total tannins (**B**), and antioxidant (**C**) profile of non-inoculated (Control), *C. rhodanensis* DK (DK), *C. rhodanensis* DK + *L. pentosus* A14-6 (DK + A14-6), and *C. rhodanensis* DK + *S. cerevisiae* TISTR 5088 (DK + TISTR 5088) during CAJ fermentation at 30 °C for seven days. Different letters (a–d) indicate significant differences in the values (*p* < 0.05). Data expressed as mean ± SD (*N* = 3).

The total phenolic content (TPC) in unfermented CAJ was 0.94 ± 0.01 mg GAE/mL. The results for unfermented control, mono-fermentation, and co-fermentation are shown in Figure 7. In this study, the TPC in CAJ fermented with *C. rhodanensis* DK exhibited an initial slight decrease during the early stages of fermentation, followed by a significant increase to 1.94 ± 0.01 mg GAE/mL after seven days of fermentation. Similarly, when CAJ was fermented with *C. rhodanensis* DK + *L. pentosus* A14-6, the TPC initially decreased and later rose significantly to 2.01 ± 0.04 mg GAE/mL. In contrast, CAJ fermented with *C. rhodanensis* DK + *S. cerevisiae* TISTR 5088 showed a consistent decline in TPC, reaching 0.39 ± 0.01 mg GAE/mL from the beginning to the end of the fermentation process. These observations lead to intriguing insights into the impact of different fermentation processes on the TPC of CAJ. Specifically, mono- and co-culture fermentation involving *C. rhodanensis* DK and *L. pentosus* A14-6 led to a significant increase in TPC compared to unfermented control CAJ. However, in contrast, the TPC of CAJ fermented with *C. rhodanensis* DK + *S. cerevisiae* TISTR 5088 displayed a notable and consistent decrease throughout the fermentation process. Kodchasee et al. [14] also reported similar findings in the context of miang tea beverages, where fermentation processes involving *C. rhodanensis* and other microorganisms resulted in increased levels of bioactive compounds. This increase in total phenolics could be attributed to microbial hydrolytic enzymes, such as polyphenol oxidase, which have the capability to degrade macropolymeric phenolic substances, thereby releasing easily detectable, free, absorbable phenolic compounds [48]. Additionally, the rise in phenolics in the system can primarily be attributed to microbial deglycosylation of glycosylated phenolic compounds from CAJ, releasing the soluble conjugated phenols or insoluble combined phenols [40]. On the other hand, the decrease in TPC observed in CAJ fermented with *C. rhodanensis* DK + *S. cerevisiae* TISTR 5088 may be attributed to the degradation of complex phenolic structures into simpler forms, such as anthocyanins, ellagic acid, flavonoids, and other phenolic derivatives [49]. The content of phenolic compounds is important to wine quality because they impact color, astringency, mouth-feel, flavor, and health benefits [50]. These findings highlight the nature of phenolic compounds during fermentation, which can be influenced by specific microorganisms for optimizing the nutritional and sensory qualities of fermented CAJ products.

#### 3.4.2. Total Tannins

The changes in total tannin content (TTC) in fermented CAJ and the control are illustrated in Figure 7. TTC in unfermented CAJ (control) was 0.43 ± 0.01 mg TAE/mL. In CAJ fermented with *C. rhodanensis* DK and *C. rhodanensis* DK + *L. pentosus* A14-6, there was an initial decrease in total tannins, followed by a gradual increase, resulting in concentrations of 1.44 ± 0.04 mg TAE/mL and 1.53 ± 0.01 mg TAE/mL, respectively. Conversely, in CAJ fermented with *C. rhodanensis* DK+ *S. cerevisiae* TISTR 5088, total tannins exhibited a different pattern. They significantly increased on day 3 of fermentation; however, after the addition of *S. cerevisiae* TISTR 5088 to CAJ containing *C. rhodanensis* DK, they notably decreased to 0.006 ± 0.002 mg TAE/mL after seven days of fermentation. Overall, the trend observed in the total tannin concentration in CAJ was influenced by the role of microorganisms.

According to the results, CAJ fermented with *C. rhodanensis* DK and *C. rhodanensis* DK + *L. pentosus* A14-6 resulted in an increase in TTC after the fermentation process. The increase in TTC aligns with the results of Kodchasee et al. [14] and Carrasco et al. [51], who reported that fermentation with yeast *C. rhodanensis* increased levels of total tannins and that *L. pentosus* was potentially involved in the metabolism of phenolic compounds. The increase in total tannins may be attributed to the breaking down of larger tannin molecules into smaller ones, as reported by Rebaya et al. [52]. Furthermore, increasing TTC contents could be attributed to microbial enzymes that break down complex phenolics and/or tannins, releasing simpler tannins that can be measured more easily as mentioned by Abdullahi et al. [25]. Moreover, during alcoholic fermentation, yeast can produce polysaccharides, which can interact with tannins, forming tannin–polysaccharide complexes. These interactions can contribute to an increase in tannin concentration, as suggested by Watrelot et al. [53]. However, the decrease in TTC observed in CAJ fermented with *C. rhodanensis* DK + *S. cerevisiae* TISTR 5088 may be attributed to the metabolism of yeast cells, especially *S. cerevisiae* and *C. rhodanensis*, or other fermentation by-products, leading to the reduction in tannins in fermented CAJ [54,55].

#### 3.4.3. Total Flavonoids

Total flavonoid content was 0.005 ± 0.002 mg QE/mL in CAJ. The content was stable after fermentation across all microbial combinations.

#### 3.4.4. Antioxidant Activity

In this study, antioxidant activity based on DPPH radical scavenging activity percentages for both fermented CAJ and the unfermented control group was evaluated throughout the fermentation process (Figure 7). The initial DPPH radical scavenging activity in the unfermented CAJ control was 53.46 ± 0.79%. However, with CAJ fermentation with *C. rhodanensis* DK, this activity significantly increased to 65.05 ± 0.22%. Similarly, when CAJ was fermented with *C. rhodanensis* DK + *L. pentosus* A14-6, the DPPH radical scavenging activity percentage increased to 62.15 ± 0.14%. On the other hand, when CAJ was fermented with *C. rhodanensis* DK + *S. cerevisiae* TISTR 5088, the DPPH radical scavenging activity increased significantly to 65.05 ± 0.34% during fermentation with initial *C. rhodanensis* DK alone. However, after the addition of *S. cerevisiae* TISTR 5088, the activity decreased to 54.63 ± 0.16% after fermentation.

This study demonstrated a substantial enhancement in the antioxidant capability of fermented CAJ when using microorganisms, namely *C. rhodanensis* DK alone and *C. rhodanensis* DK along with *L. pentosus* A14-6. The observed increase in DPPH radical scavenging activity was statistically significant (*p* < 0.05) compared to the control group. The improved antioxidant activity observed in this experiment is likely linked to the accumulation of antioxidant molecules, including phenolics and tannins. Many other polyphenols such as flavonols, anthocyanins, related phenolic oligomers, and phenolic acids are known for their diverse biological activities, primarily attributed to their potent antioxidant properties [56]. Additionally, bioactive compounds like condensed tannins or proanthocyanidins, which are responsible for imparting astringency in certain fruits, were found to exhibit robust radical scavenging activity [57]. Furthermore, the increase in antioxidant activity may be attributed to yeast cells, which play a role in defending against oxidative stress by producing antioxidant compounds like glutathione and ergothioneine during fermentation [58]. On the other hand, the co-fermentation of *C. rhodanensis* DK+ *S. cerevisiae* TISTR 5088 did not result in a significant difference in antioxidant activity after fermentation. This observation may be linked to the accumulation of bioactive compounds as mentioned above, where *S. cerevisiae* may be associated with reducing total phenolics and therefore modifying the antioxidant capacity of fermented CAJ [55]. These findings underscore the potential health benefits of fermented CAJ and the importance of selecting specific microorganism combinations to optimize its antioxidant properties.

### 3.5. β-Glucosidase Activity

**Figure 8:**
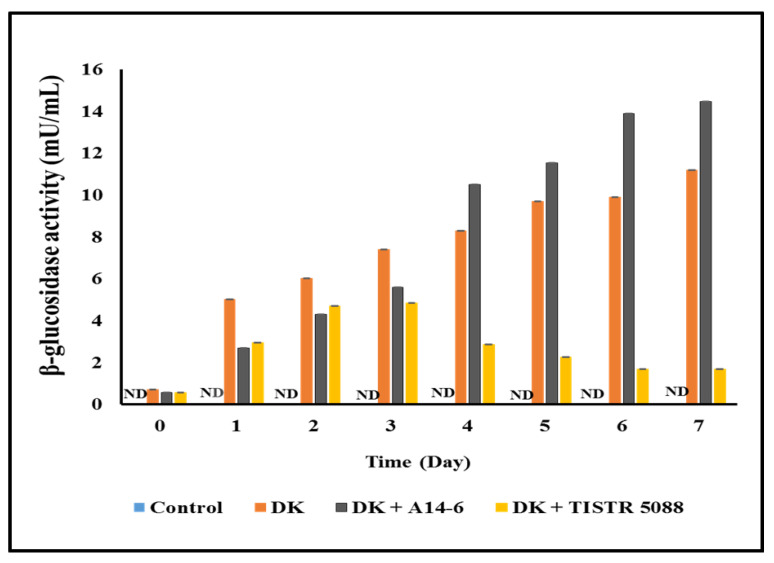
β-Glucosidase activity of non-inoculated (Control), *C. rhodanensis* DK (DK), *C. rhodanensis* DK + *L. pentosus* A14-6 (DK + A14-6), and *C. rhodanensis* DK + *S. cerevisiae* TISTR 5088 (DK + TISTR 5088) during CAJ fermentation at 30 °C for seven days. ND indicates that no enzyme activity was detected. Data expressed as mean ± SD (*N* = 3).

In this study, the investigation of β-glucosidase activity was carried out in fermented CAJ. In the context of mono-fermentation, the enzyme activity increased to 11.18 ± 0.04 mU/mL during the fermentation period. However, in the co-culture fermentation, a different trend emerged. Enzyme activity was observed at 1.67 ± 0.01 mU/mL, indicating a decrease when *C. rhodanensis* DK was combined with *S. cerevisiae* TISTR 5088. Conversely, when *C. rhodanensis* DK was combined with *L. pentosus* A14-6, the enzyme activity showed an increase, reaching 14.45 ± 0.02 mU/mL after fermentation. These enzyme activity results are visually presented in Figure 8. The observed variations in enzyme activity between mono-fermentation and co-fermentation highlight the dynamic nature of microbial interactions during the fermentation process. In mono-fermentation, enzyme activity increased, indicating that *C. rhodanensis* DK had a positive impact on enzyme production [18]. This result aligns with the findings of Kodchasee et al. [14], who studied *C. rhodanensis* isolated from miang and found its capability of β-glucosidase production during the fermentation process of low-alcoholic miang wine. However, when co-cultured with *S. cerevisiae* TISTR 5088, a decrease in enzyme activity was observed. This decrease could be attributed to various factors, including competition for resources or differences in metabolic pathways between the two microorganisms, as suggested by Rowland et al. [59]. In contrast, the combination of *C. rhodanensis* DK with *L. pentosus* A14-6 resulted in a substantial increase in enzyme activity. This suggests a potential synergistic interaction between these microorganisms, where they complement each other’s metabolic activities. Such interactions can lead to enhanced enzyme production and overall improvement in the fermentation performance. Furthermore, *L. pentosus* was found to be positive for β-glucosidase enzyme production according to the study by Lorn et al. [60]. Further research is warranted to elucidate the underlying mechanisms behind these microbial interactions and their impact on enzyme production. Additionally, exploring how these dynamics contribute to the sensory attributes of the fermented product is also an area that requires further investigation.

### 3.6. Determination of In Vitro α-Glucosidase Inhibitory Activity

**Figure 9:**
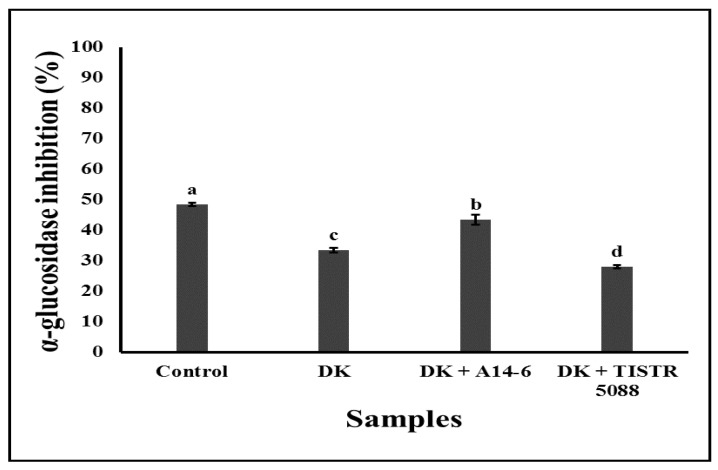
In vitro α-glucosidase inhibitory activity of non-inoculated (Control), *C. rhodanensis* DK (DK), *C. rhodanensis* DK + *L. pentosus* A14-6 (DK + A14-6), and *C. rhodanensis* DK + *S. cerevisiae* TISTR 5088 (DK + TISTR 5088) after CAJ samples were fermented at 30 °C for seven days. Different letters (a–d) indicate significant differences in the values (*p* < 0.05). Data expressed as mean ± SD (*N* = 3).

α-Glucosidase inhibitors can competitively inhibit the activity of small intestinal α-glucosidase and delay or inhibit the absorption of glucose in the small intestine, preventing elevation of the postprandial blood glucose level, and therefore, play a significant role as chemotherapeutic agents for noninsulin-dependent diabetes mellitus [61]. This study investigated in vitro α-glucosidase inhibitory activities of the fermented CAJ against α-glucosidase from *S. cerevisiae*. In this study, we used yeasts, especially *S. cerevisiae*, in co-culture fermentation, as reported by Ahmed et al. [29]. As mentioned in these studies, *S. cerevisiae* can efficiently produce α-glucosidase, and therefore the samples were incubated at a temperature ranging from 30–80 °C to deactivate the α-glucosidase enzyme inside the samples. Among different temperatures, samples incubated at 70 °C showed the highest α-glucosidase inhibitory activity. The data of the highest α-glucosidase inhibitory activity of CAJ and fermented CAJ deactivated at 70 °C are shown in Figure 9. According to the result, unfermented CAJ displayed α-glucosidase inhibitory activity of 48.51 ± 0.51%. However, when CAJ was fermented with *C. rhodanensis* DK, the inhibitory activity decreased to 33.50 ± 0.76%. Remarkably, when CAJ was fermented with *C. rhodanensis* DK + *L. pentosus* A14-6, the activity slightly decreased to 43.46 ± 1.71%. Further, when CAJ was fermented with *C. rhodanensis* DK + *S. cerevisiae* TISTR 5088, α-glucosidase inhibitory activity decreased to 28.07 ± 0.51%.

Our findings revealed that fermentation with various microbial combinations generally resulted in decreased inhibitory activity compared to control. Interestingly, the co-culture fermentation with *C. rhodanensis* DK + *L. pentosus* A14-6 displayed the highest residual inhibitory activity among fermented CAJ samples, even though there was a slight decrease compared to the control. This decrease could be attributed to the depletion of inhibitory compounds, especially fructose present in the unfermented CAJ. Alcoholic fermentation is the process of conversion of monosugars, especially glucose, and fructose, in CAJ to ethanol. Fructose has been reported to possess inherent α-glucosidase inhibitory activity [62]. Furthermore, the varying inhibitory activities among the fermented samples suggest the potential contribution of other factors such as phenolic compounds, which are known α-glucosidase inhibitors [63], in the presence of *L. pentosus*, which has inherent inhibitory activity towards the enzyme α-glucosidase [64]. Further, the lower inhibitory activities in mono- and co-culture fermentation of *C. rhodanensis* DK and *C. rhodanensis* DK + *S. cerevisiae* TISTR 5088 compared to co-culture fermentation with *C. rhodanensis* DK + *L. pentosus* A14-6 could be related to the specific metabolic pathways of the microorganisms employed in fermentation influencing the production or degradation of α-glucosidase inhibitory compounds [65].

### 3.7. Comparison of Fermented CAJ Product Properties Using Different Microbial Inoculums

The overall physico-chemical characteristics of the fermented CAJ samples using different microbial inoculums are presented in Table 1. The type of microbes used and their metabolic activities significantly influence the accumulation of various metabolites such as organic acids, ethanol, and some phenolic compounds, leading to changes in the final product properties. A significant decrease in pH and an increase in total titratable acidity were observed across all fermented samples, especially in the co-culture fermentation with *C. rhodanensis* DK + *L. pentosus* A14-6. Corresponding to the lower pH, lower sugar concentration was also observed. In addition, a significant increase in the bioavailability of phenolic compounds, particularly the content of total polyphenols and tannins, particularly the product fermented with *C. rhodanensis* DK + *L. pentosus* A14-6 showed the most substantial increase in total polyphenols and total tannins. Consistent with the rise in total polyphenols and tannins, the antioxidant activity (DPPH scavenging activity) also increased significantly after fermentation. Although microbial fermentation revealed a decrease in α-glucosidase inhibitory activity, an indication of potential blood sugar control, in all fermented samples compared to the control, the co-culture fermentation with *C. rhodanensis* DK + *L. pentosus* A14-6 retained a significantly higher level of inhibitory activity (43.5%) among fermented products. The enhanced levels of phenolic compounds and antioxidants indicate significant health benefits, including reducing the risk of chronic diseases such as heart disease, cancer, and inflammatory disorders, while antioxidant activity can help protect cells from damage caused by free radicals [64]. A complete or near-complete sugar conversion in the fermentation process also provides a desirable final product for individuals managing their overall sugar intake. The increase in antioxidants in fermented CAJ can provide a rich source of health-protective compounds and may be targeted as high-value antioxidant nutraceutical inhibitors relevant as antidotes to oxidative stress-linked non-communicable diseases (NCDs) such as type 2 diabetes [4]. The data from this study strongly support the potential use of *C. rhodanensis* DK in the fermentation of CAJ, particularly in collaboration with *L. pentosus* A14-6. Therefore, a co-culture fermentation of CAJ with *C. rhodanensis* DK + *L. pentosus* A14-6 presents a strategy for creating fermented CAJ potentially rich in health-promoting bioactive compounds with antidiabetic properties and a suitable ethanol content for low-alcohol beverage applications.

## 4. Conclusions

The optimal sugar concentration for *C. rhodanensis* DK fermentation was found to be 5% (*w*/*v*) and 10% (*w*/*v*) glucose and the optimal CAJ composition was at pH adjusted to 6. All parameters showed better activities in co-culture fermentation compared to mono-culture fermentation in this analysis. The co-fermentation of *C. rhodanensis* DK with *L. pentosus* A14-6 resulted in a higher sugar consumption rate than monoculture, the highest production of total titratable acidity, the highest bioactive compound levels, and high antioxidant activity, and also showed the highest percent of α-glucosidase inhibitory activity. On the other hand, co-fermentation with *S. cerevisiae* TISTR 5088 resulted in the highest sugar consumption rate, although it showed lower enzyme production, bioactive compounds, antioxidant activity, and α-glucosidase inhibitory activity. The findings of this study emphasize the significance of co-culture fermentations and reveal their potential to enhance various parameters compared to mono-culture fermentations. The increased sugar consumption rates and the elevated production of bioactive compounds in co-culture fermentations with *L. pentosus* A14-6 suggest a synergistic interaction between the microorganisms, contributing to a better healthy and metabolically active fermentation process compared to the use of mono-culture fermentation with *C. rhodanensis* DK alone. Low-alcohol beverage production as reported in this study is not only helpful to remove the sugars that can negatively influence health, especially in diabetic patients, but also enhances the bioactive compounds and antioxidant activity. Furthermore, a significant percentage of α-glucosidase inhibitory activity was also retained, which confers potential anti-glycemic benefits. Therefore, this fermented CAJ can be regarded as a low-alcohol, healthy beverage for consumers. These findings have important implications for the food and beverage industries.

## Figures and Tables

**Table 1:** Comparison of fermented CAJ product properties achieved from different microbial inoculums.

Parameters	Control	*C. rhodanensis* DK	*C. rhodanensis* DK + *L. pentosus* A14-6	*C. rhodanensis* DK + *S. cerevisiae* TISTR 5088
pH	6.05	5.61	3.58	5.09
Total titratable acidity (M)	0.02 ± 0.01 ^d^	0.04 ± 0.01 ^b^	0.28 ± 0.01 ^a^	0.03 ± 0.01 ^c^
Total sugars (g/L)	68.52 ± 0.01 ^a^	7.71 ± 0.01 ^b^	4.96 ± 0.02 ^c^	2.33 ± 0.02 ^d^
Reducing sugars (g/L)	64.10 ± 0.00 ^d^	6.70 ± 0.03 ^c^	3.04 ± 0.03 ^b^	1.82 ± 0.01 ^a^
Type of sugars (g/L)	G* 30.13 ± 0.14 ^a^	G* 1.39 ± 0.04 ^b^	G* nd.*	G* nd.*
	F* 37.49 ± 0.15 ^a^	F* 5.21 ± 0.16 ^b^	F* 3.20 ± 0.05 ^c^	F* 0.24 ± 0.03 ^d^
	S* 2.13 ± 0.03 ^a^	S* 2.15 ± 0.03 ^a^	S* 2.12 ± 0.03 ^a^	S* nd.*
Ethanol (g/L)	nd.*	28.23 ± 0.16 ^b^	19.47 ± 0.06 ^c^	33.61 ± 0.11 ^a^
Total polyphenols (mg GAE/mL)	0.94 ± 0.01 ^b^	1.94 ± 0.01 ^a^	2.01 ± 0.04 ^a^	0.39 ± 0.01 ^c^
Total tannins (mg TAE/mL)	0.43 ± 0.01 ^c^	1.44 ± 0.04 ^b^	1.53 ± 0.01 ^a^	nd.*
Antioxidant activity (% DPPH scavenging)	53.46 ± 0.79 ^d^	65.05 ± 0.34 ^a^	62.14 ± 0.34 ^b^	54.63 ± 0.16 ^c^
α-Glucosidase inhibitory activity (%)	48.51 ± 0.51 ^a^	33.50 ± 0.76 ^c^	43.46 ± 1.71 ^b^	28.07 ± 0.51 ^d^

* Note: The letters represent (G) glucose, (F) fructose, and (S) sucrose. Different letters (a–d) indicate significant differences in the values (*p* < 0.05). nd. = none detectable. Data expressed as mean ± SD (*N* = 3).

## Data Availability

The original contributions presented in the study are included in the article, further inquiries can be directed to the corresponding author.
